# Chitosan-modified liposomes for improved oral delivery of dihydromyricetin: synergistic stabilization, pH-responsive release, and markedly enhanced bioaccessibility

**DOI:** 10.1016/j.fochx.2026.104259

**Published:** 2026-07-30

**Authors:** Hui Teng, Minyi Yan, Chong Chen, Chao Ai, Huangjuan Huo, Lei Chen

**Affiliations:** College of Food Science and Technology, Guangdong Ocean University, Guangdong Provincial Key Laboratory of Aquatic Product Processing and Safety, Guangdong Province Engineering Laboratory for Marine Biological Products, Guangdong Provincial Engineering Technology Research Center of Seafood, Guangdong Provincial Engineering Technology Research Center of Prefabricated Seafood Processing and Quality Control, Zhanjiang 524088, China

**Keywords:** Dihydromyricetin, Chitosan, Liposomes, *In vitro* release, Gastrointestinal digestion

## Abstract

In this study, chitosan-modified dihydromyricetin liposomes (CHI-DMY-Lips) were developed *via* electrostatic adsorption to overcome the low bioavailability of dihydromyricetin (DMY). Employing conventional DMY liposomes (DMY-Lips) as controls, a comprehensive assessment was conducted on the physicochemical properties, activity preservation, *in vitro* release kinetics, as well as the structural stability and bioaccessibility during simulated gastrointestinal (GI) digestion of the delivery system. CHI-DMY-Lips exhibited a positive surface charge with larger size, more uniform distribution, and higher encapsulation efficiency (54.00% compared to 41.01% in DMY-Lips). The encapsulation by liposomes preserved the antioxidant activity of DMY, whereas CHI further augmented its activity through synergistic effects. CHI-DMY-Lips presented enhanced structural and electrostatic stability during storage and superior *in vitro* sustained-release performance (especially at pH 7.4). Besides, CHI-DMY-Lips retained structural integrity throughout GI digestion and preserved its effective concentration and activity, resulting in a bioaccessibility approximately threefold that of free DMY (25.97% *vs.* 9.73%).

## Highlights

• Chitosan-modified DMY liposomes showed higher encapsulation efficiency.
• Chitosan boosts DMY's antioxidant activity through synergistic interactions.
• Chitosan-modified liposomes presented improved stability and sustained release.
• Chitosan-modified liposomes remained intact in simulated gastrointestinal digestion.
• Chitosan-modified liposomes achieved 2.67× DMY bioaccessibility *vs* free form (9.73%).

## 1 Introduction

The oral delivery of hydrophobic bioactive compounds remains a serious challenge in functional food development and pharmaceutical applications. DMY, a natural flavonoid from plants such as *Ampelopsis grossedentata*, serves as a representative example. Despite the demonstrated antioxidative, anti-inflammatory, glycolipid metabolism regulatory, and hepatoprotective effects of DMY, its therapeutic potential is severely restricted by multiple factors (Hou et al., 2025). These include poor aqueous solubility, limited chemical stability within the GI tract, and vulnerability to degradation or transformation under diverse pH conditions, as well as the influence of digestive enzymes and microbial activity (Chen et al., 2020; Hou et al., 2025). Collectively, these factors lead to its low oral bioavailability (≤15%).

Various encapsulation systems, including polymeric nanoparticles, cyclodextrin complexes, and lipid-based carriers, have been explored for the oral delivery of phenolic compounds; among them, liposomes offer unique advantages in terms of biocompatibility and membrane-mimetic properties (Hu et al., 2025; Kasapoglu et al., 2024). Liposomes possess a phospholipid bilayer structure that can encapsulate both hydrophilic and hydrophobic substances, effectively improving their solubility and stability. Accordingly, liposomes represent a promising strategy for enhancing the stability and absorption of DMY (Guimaraes et al., 2021). Nevertheless, conventional liposomes suffer from inherent limitations: they are susceptible to disruption by bile salts and enzymes, exhibit physical instability (such as aggregation, fusion, and premature drug leakage), and have a short residence time in the GI tract, all of which collectively reduce the delivery efficiency (Ding et al., 2024). To address these shortcomings, surface modification with natural biopolymers has emerged as a viable approach. Chitosan (CHI), a mucoadhesive and biodegradable polymer, can electrostatically coat liposomes to form a protective barrier, thereby improving colloidal stability and enabling pH-responsive release (Abd El-Hack et al., 2020; Bhaskaran et al., 2022).

Although CHI-modified liposomes have been studied for various bioactive compounds, research on DMY-loaded CHI-modified liposomes is limited, especially the potential of these functionalized systems for DMY encapsulation remains underexplored. Ding et al. (2024) recently reported CHI-modified DMY liposomes for liver injury treatment, but their work mainly focused on *in vivo* efficacy. Our study differs by providing a systematic evaluation of formulation characteristics, including physicochemical properties, release behavior, and GI digestion stability, which have not been fully addressed in previous studies. Consequently, this study was intended to develop and systematically evaluate two DMY-loaded liposomal formulations, namely conventional liposomes and CHI-modified liposomes. The research goals were as follows: (1) To characterize the efficacy of the CHI coating and molecular interactions *via* ultraviolet-visible (UV–Vis) absorption and Fourier transform infrared (FTIR) spectroscopy; (2) To evaluate the morphology, colloidal stability, and antioxidant capacity; (3) To quantify the pH-dependent release profiles and model the release kinetics; (4) To assess the digestive stability and bioaccessibility by utilizing a standardized *in vitro* GI model. This work establishes CHI-DMY-Lips as a promising oral delivery platform, offering fundamental insights for the applications of DMY in functional foods and therapeutics.

## 2 Materials and methods

### 2.1 Materials and chemicals

Soybean L-*α*-phosphatidylcholine (PC) was purchased from Sigma-Aldrich Chemical Co. (St. Louis, MO, USA). DMY (>98% purity), 2,2-diphenyl-1-picrylhydrazyl (DPPH), and 2,2′-azino-bis (3-ethylbenzothiazoline-6-sulfonic acid) diammonium salt (ABTS) powders were purchased from Shanghai Aladdin Bio-Chem Technology Co., Ltd. (Shanghai, China). Phosphate-buffered saline (PBS), acetic acid solution (1%), *α*-amylase (40–60 U/mg), pepsin (> 3000 U/mg), trypsin (250 U/mg), and bile salts were purchased from Shanghai Yuan Ye Biotechnology Co., Ltd. (Shanghai, China). CHI and acarbose were purchased from Shanghai Macklin Biochemical Co., Ltd. (Shanghai, China). Anhydrous ethanol was purchased from Guangzhou Guanghua Technology Co., Ltd. (Guangzhou, China). All other chemicals used were of analytical grade.

### 2.2 Preparation of DMY-Lips and CHI-DMY-Lips

DMY-Lips were prepared based on the method of Chen et al. (2022). Briefly, 10 mL of a chloroform solution containing PC (40 mg) was thoroughly mixed with 5 mL of an ethanol solution containing DMY (15 mg). Then, the solvent was removed using a rotary evaporator in a 30 °C water bath under reduced pressure (100 mbar) at a rotation speed of 100 r/min, until a uniform lipid film was formed. Subsequently, the film was exposed to a high-purity nitrogen flow for 5 min to completely remove the residual solvent. After that, 20 mL of PBS (10 mmol/L, pH 7.4) was added to the flask. The mixture was vortex-mixed for 2 min and then subjected to ultrasonic treatment for 2 min to facilitate hydration. The resulting liposomal mixture was filtered three times through a polyethersulfone membrane (pore size: 0.22 μm) to obtain a unilamellar liposome suspension. CHI-DMY-Lips were prepared following a slightly modified method from Imam et al. (2021). A CHI solution (0.5 mg/mL) was magnetically stirred with DMY-Lips in a 1:1 ratio for 1 h. After that, the resulting mixture was filtered three times through a polyethersulfone membrane (pore size: 0.45 μm) to yield a bilamellar liposome suspension and stored at 4 °C for further analysis. In addition, CHI-modified liposomes without DMY (termed "CHI-Blank-Lips") were prepared according to the aforementioned method for comparative studies.

### 2.3 Encapsulation efficiency and apparent drug loading capacity

The encapsulation efficiency (EE) of the liposomal samples was determined by centrifugal extraction and calculated according to the method of Wang et al. (2026). Aliquots (2 mL per sample) of the liposome suspensions (DMY-Lips and CHI-DMY-Lips) were centrifuged at 10000 r/min for 20 min at 4 °C to precipitate the liposome pellets and separate the free (unencapsulated) DMY in the supernatant. Subsequently, 1 mL of the supernatant was extracted, diluted appropriately with PBS (10 mmol/L, pH 7.4), and its absorbance at 290 nm was measured using a UV–Vis spectrophotometer (Cary 60 UV–Vis, Agilent Technologies Inc., USA). The concentration of free DMY was calculated using a standard calibration curve (*y* = 0.0392*x* + 0.0102, R^2^ = 0.999), which was established with DMY standard solutions in the concentration range of 5 to 12 μg/mL. EE was determined using Eq. (1):

(1) $$\mathrm{EE}\left(\%\right)=\left(m_{\text{total}}-m_{\text{free}}\right)/m_{\text{total}}\times 100$$

where m_total_ represents the total amount of DMY initially added during liposome preparation (15 mg), and m_free_ denotes the amount of DMY in the free form determined from the supernatant.

In addition, the apparent drug loading capacity (DLC_app_) was theoretically estimated based on the input masses of the carrier materials and the experimentally obtained EE values, using the following equations:

For DMY-Lips:

(2) $${\mathrm{DLC}}_{\mathrm{app}}\left(\%\right)=m_{\mathrm{DMY}}\times \mathrm{EE}/\left(m_{\mathrm{PC}}+m_{\mathrm{DMY}}\times \mathrm{EE}\right)\times 100$$

For CHI-DMY-Lips:

(3) $${\mathrm{DLC}}_{\mathrm{app}}\left(\%\right)=m_{\mathrm{DMY}}\times \mathrm{EE}/\left(m_{\mathrm{CHI}}+m_{\mathrm{PC}}+m_{\mathrm{DMY}}\times \mathrm{EE}\right)\times 100$$

where m_DMY_ is 15 mg, m_PC_ is 40 mg, and m_CHI_ is 10 mg. Notably, the calculated DLC_app_ for CHI-DMY-Lips represents a theoretical minimum (apparent value), as the exact amount of CHI actually adsorbed onto the liposome surface was not experimentally quantified in this study.

### 2.4 Measurement of liposomal size and charge

The liposomal samples (DMY-Lips and CHI-DMY-Lips, 1 mL of each) were diluted in PBS (10 mmol/L, pH 7.4) at a ratio of 1:5 and subjected to analyses of average particle size, zeta potential, and polydispersity index (PDI) at 25 °C by dynamic light scattering (DLS) measurements (Zetasizer Nano-ZSE, Malvern Instruments, U.K.).

### 2.5 Morphological observation

For visual inspection, the liposomal samples (DMY-Lips and CHI-DMY-Lips) were placed in transparent glass vials and photographed for macroscopic observation. For transmission electron microscope (TEM) analysis, the morphology and particle size distribution of the liposomal samples were examined using a TEM (JEM 1400 Plus, JEOL Ltd., Japan). The liposomal solutions were diluted tenfold with ultrapure water, and a droplet of each sample was applied to a copper grid. Subsequently, the samples were stained with a uranyl acetate solution (2%). After air-drying, the copper grid was positioned in the TEM for detailed observation and image acquisition of the microstructure.

### 2.6 UV–Vis absorption spectroscopy

The UV–Vis absorption spectra of the liposomal samples (DMY-Lips and CHI-DMY-Lips) and the control samples (DMY and CHI-Blank-Lips) were acquired using a UV–Vis spectrophotometer (Cary 60 UV–Vis, Agilent Technologies Inc., USA) over the range of 200 to 800 nm.

### 2.7 FTIR spectroscopy

FTIR spectra of the liposomal samples (DMY-Lips and CHI-DMY-Lips) were recorded using an FTIR spectrometer (TENSOR 27, Bruker Optics Inc., Germany) across the range of 4000 to 400 cm^−1^, following the method described by Chen et al. (2022). For comparative purposes, the spectra of DMY, CHI, and CHI-Blank-Lips were also acquired under the same conditions.

### 2.8 Assessment of *in vitro* antioxidant activities

#### 2.8.1 DPPH radical scavenging assay

The antioxidant activities of the liposomal samples (DMY-Lips and CHI-DMY-Lips) were initially assessed using the DPPH radical scavenging assay, as described by Tan et al. (2014). Briefly, 2.958 mg of DPPH was dissolved in anhydrous ethanol to obtain a final volume of 50 mL, resulting in a 0.15 mmol/L DPPH solution. Then, 2 mL of each sample at different concentrations (25, 50, and 100 μg/mL) was combined with 2 mL of the DPPH ethanol solution. Following a 30-min reaction in the dark, the absorbance was measured at 517 nm using the aforementioned UV–Vis spectrophotometer. Subsequently, the DPPH radical scavenging activity was calculated according to Eq. (4):

(4) $$\text{DPPH radical scavenging activity}\;\left(\%\right)=\left[1-\left(A₂-A₁\right)/A₀\right]\times 100$$

where A₀ represents the absorbance of the control (2 mL DPPH solution + 2 mL ethanol), A₁ signifies the absorbance of the sample background (2 mL sample + 2 mL ethanol), and A₂ denotes the absorbance of the test sample (2 mL sample + 2 mL DPPH solution).

#### 2.8.2 ABTS radical scavenging assay

ABTS radical scavenging activity was determined based on the procedure from Zheng et al. (2016) with slight modifications. The ABTS radical cation (ABTS•^+^) was generated by mixing an equal volume of ABTS solution (7 mmol/L) with a potassium persulfate solution (2.45 mmol/L), followed by incubation of the mixture in the dark at room temperature for 12–16 h. Before use, the ABTS working solution was diluted with PBS (50 mmol/L, pH 7.4) to achieve an absorbance of 0.70 ± 0.02 at 734 nm. Then, 50 μL of the sample at different concentrations (25, 50, and 100 μg/mL) was added to 150 μL of the diluted ABTS working solution and incubated at 30 °C for 30 min. The absorbance at 734 nm was measured using a microplate reader (Varioskan LUX, Thermo Fisher Scientific, USA). A blank control was prepared by replacing the sample with an equal volume of PBS (50 mmol/L, pH 7.4). The ABTS•^+^ scavenging activity was calculated based on Eq. (5):

(5) $$\text{ABTS}•{}^{+}\;\text{scavenging activity}\;\left(\%\right)=\left(A_{\text{Control}}-A_{\text{Sample}}\right)/A_{\text{Control}}\times 100$$

where A_Control_ denotes the absorbance of the blank control group, and A_Sample_ represents the absorbance of the sample group.

### 2.9 Storage stabilities

The liposomal samples (DMY-Lips and CHI-DMY-Lips) were aliquoted into sample vials and stored at 4 °C and 25 °C for 0, 5, 10, 15, 20, 25, and 30 days, respectively. At each time point, three vials were analyzed for EE, particle size, zeta potential, and PDI, and average values were calculated to assess the storage stability of the liposomal samples.

### 2.10 Assessment of *in vitro* release

The *in vitro* release characteristics of the liposomal samples (DMY-Lips and CHI-DMY-Lips) were evaluated using a balanced dialysis method, with DMY serving as the control. The procedure entailed reconstituting 2 mL of PBS (10 mmol/L, pH 7.4) with lyophilized liposomal samples, maintaining a consistent DMY concentration. The mixture was then placed into a clean, activated dialysis bag (molecular weight cutoff 8000–14,000 Da). The bag was tightly tied to prevent any leakage. It was subsequently immersed in a centrifuge tube containing 30 mL of release medium (PBS containing 0.05% Tween 80, 10 mmol/L, with pH values of 1.2, 6.8, and 7.4, respectively). Incubation took place in a constant-temperature water bath shaker at 37 °C and 100 r/min for 96 h. Samples of 1 mL were taken at 0.5, 1, 2, 4, 6, 8, 10, 12, 24, 48, 72, and 96 h. After each sampling, 1 mL of fresh release medium was added as a replacement.

The quantity of DMY in the release medium was determined *via* UV–Vis spectrophotometry to compute the cumulative release rate (Q). By plotting the sampling time on the *x*-axis and cumulative DMY release on the *y*-axis, release curves at different pH levels were generated, and the drug release rates at each sampling time point were calculated using the following Eq. (6):

(6) $$Q\;\left(\%\right)=\left(\mathrm{CnV}+\sum^{n-1}\text{CiVi}\right)/m\times 100$$

In this equation, Cn denotes the concentration of DMY in the release medium at the n^th^ sampling (mg/mL). Similarly, Ci denotes the concentration of DMY in the release medium at the i^th^ sampling (mg/mL). V stands for the total volume of the release medium (mL), while Vi represents the volume of the i^th^ sample (mL). Lastly, m signifies the total mass of DMY in the sample (mg).

### 2.11 *In vitro* simulated digestion evaluation

#### 2.11.1 *In vitro* simulated digestion

The simulated salivary fluid (SSF), simulated gastric fluid (SGF), and simulated intestinal fluid (SIF) were prepared, and the digestion procedures were carried out according to the methods of Brodkorb et al. (2019) and Minekus et al. (2014). An *in vitro* three-stage simulated model (oral-gastric-intestinal) was established to systematically assess the digestive behavior and stability of DMY, DMY-Lips, and CHI-DMY-Lips. The simulation procedure was conducted as follows: To simulate oral digestion, the SSF was mixed with the samples, and *α-*amylase (with a final concentration of 75 U/mL in the mixture) was added to the mixture. Subsequently, the pH value was adjusted to 7.0, and the mixture was incubated for 2 min at 37 °C at a shaking speed of 100 rpm in a water-bath shaker. For the gastric digestion stage, the orally digested sample and the SGF were mixed at a volume ratio of 1:1. Pepsin was added to the mixture to reach a final concentration of 2000 U/mL, and the pH was adjusted to 3.0. After incubation for 120 min (37 °C, 100 rpm in a water-bath shaker), the gastric-digested sample was mixed with the SIF at a volume ratio of 1:1. Pancreatin (with a pancreatin activity of 100 U/mL in the final mixture) and bile salts (with a final concentration of 10 mM in the mixture) were added, and the pH of the mixture was adjusted to 7.0. This mixture was then incubated for 120 min (37 °C, 100 rpm in a water-bath shaker) to simulate small intestinal digestion.

#### 2.11.2 Multi-index dynamic monitoring of the digestion process

At each stage of the digestion process, DLS was employed to quantify the variations in particle size, PDI, and zeta potential, aiming to assess the impacts of the digestion process on the physicochemical properties of the liposomal samples. Meanwhile, the morphology of liposomes was observed using optical microscopy. Additionally, samples were collected (at the oral digestion stage, as well as the intermediate and final stages of gastric and small intestinal digestion) to determine the changes in the concentration of DMY and the DPPH radical scavenging capacity according to the aforementioned methods.

#### 2.11.3 Determination of bioaccessibility

The bioaccessibility of DMY was determined according to the method of Hu et al. (2023). After the intestinal digestion stage was completed, the raw digestive juice was collected and centrifuged at 12000 rpm and 4 °C for 10 min. The supernatant, serving as the micellar phase and containing soluble DMY, was mixed with absolute ethanol at a 1:1 (*v*/v) ratio and subjected to vortex extraction. Subsequently, centrifugation was carried out at 12000 rpm and 4 °C for 10 min. The upper extraction layer was carefully collected. DMY content was measured using a UV–Vis spectrophotometer, and its bioaccessibility was calculated according to the following formula (7):

(7) $$\text{Bioaccessibility}=C_{\text{Micelles}}/C_{\text{Initial}}\times 100\%$$

In this equation, C_Micelles_ and C_Initial_ represent the concentration of DMY in the micellar phase and the initial sample, respectively.

### 2.12 Statistical analysis

Results were presented as "means ± standard deviations (SD)" of at least triplicate independent determinations. Statistical analysis was performed using OriginPro 2025b (OriginLab Corp., Northampton, USA). One-way analysis of variance (ANOVA) was conducted, and significant differences between group means were determined using Tukey's post-hoc test at the significance level of *p* < 0.05.

## 3 Results and discussion

### 3.1 EE, DLC_app_, particle size, PDI, zeta potential, and morphological analysis

EE is a crucial indicator of liposome quality, reflecting the retention capability of encapsulated active substances and assessing whether the nanoparticles can carry sufficient amounts of bioactive compounds to achieve their desired health benefits (Ding et al., 2024). The EE values for DMY-Lips and CHI-DMY-Lips were determined to be 41.01 ± 2.09% and 54.00 ± 0.29%, respectively (Table 1). The lower EE of DMY-Lips compared to the 88.19% reported by Ding et al. (2024)—despite a similar thin-film hydration method—may be attributed to two factors. First, their EE calculation excluded drug lost or degraded during preparation, whereas our value is based on total initial DMY input. Second, the absence of cholesterol in our formulation may have further reduced EE, as cholesterol is known to intercalate between phospholipid molecules, reduce membrane fluidity, and enhance the structural integrity of the lipid bilayer, thereby minimizing drug leakage during preparation (Kaddah et al., 2018). Furthermore, the increased EE observed in CHI-DMY-Lips can potentially be ascribed to CHI sealing the surface pores of the liposome membrane, thereby inhibiting DMY leakage (Esposto et al., 2021). In contrast, EE slightly decreased (from 88.19% to 86.57%) upon CHI coating in the cholesterol-stabilized system of Ding et al. (2024), suggesting that the protective effect of CHI is more pronounced in less rigid membranes, where it can seal structural defects more effectively.

**Table 1:** The physicochemical properties of dihydromyricetin liposomes (DMY-Lips) and chitosan-modified dihydromyricetin liposomes (CHI-DMY-Lips).^a^

Sample	EE (%)	DLC_app_ (%)^b^	Particle size (nm)	PDI	Zeta potential (mV)
DMY-Lips	41.01 ± 2.09^b^	13.33	171.04 ± 2.91^b^	0.24 ± 0.01^a^	−19.80 ± 0.90^b^
CHI-DMY-Lips	54.00 ± 0.29^a^	13.94	378.10 ± 10.13^a^	0.18 ± 0.02^a^	+23.50 ± 1.02^a^

a EE refers to encapsulation efficiency; DLC_app_ refers to the apparent drug loading capacity; PDI refers to polydispersity index. Values are presented as mean ± standard deviation (SD) (n = 3). Means in the same column with different lowercase letters indicate significant differences (p < 0.05, one-way analysis of variance (ANOVA) followed by Tukey's post-hoc test).

b DLC_app_ values are theoretical estimates calculated from the mean EE values; thus, standard deviations are not determined (n.d.) for this calculated parameter.

In addition to EE, the DLC_app_ was calculated to be 13.33% for DMY-Lips and 13.94% for CHI-DMY-Lips (Table 1). Although the CHI coating introduced additional mass to the carrier system, the CHI-DMY-Lips exhibited a slightly higher DLC_app_ than DMY-Lips. This increase is attributed to the substantial improvement in EE (from 41.01% to 54.00%), which effectively compensated for the mass of the CHI polymer. It should be noted that the DLC_app_ of CHI-DMY-Lips is a theoretical value based on the total added CHI; the true experimental DLC may differ depending on the actual amount of CHI bound to the liposomal surface, which warrants further investigation *via* freeze-drying and gravimetric analysis in future studies.

**Fig. 1:**
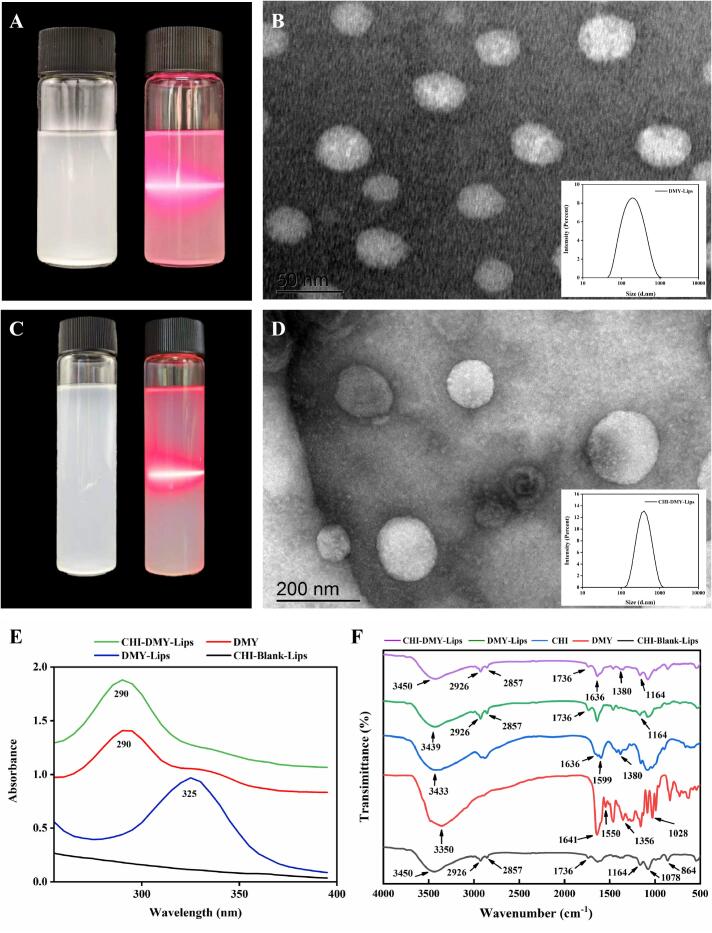
Characterization of dihydromyricetin (DMY) liposomes (DMY-Lips) and chitosan-modified DMY liposomes (CHI-DMY-Lips). (A, B) Photographic images and transmission electron microscopy (TEM) images (with particle size distribution) of DMY-Lips; (C, D) Corresponding images of CHI-DMY-Lips; (E) Ultraviolet-visible (UV–Vis) absorption spectra of free DMY and the two liposomal formulations; (F) Fourier transform infrared (FTIR) spectra of chitosan (CHI), free DMY, DMY-Lips, and CHI-DMY-Lips.

The particle size of liposomes is a critical parameter in evaluating their performance. For medical applications, the dimensions of liposomes typically range from 50 nm to 450 nm (Etheridge et al., 2013). As shown in Table 1, the average particle sizes of DMY-Lips and CHI-DMY-Lips were 171.04 ± 2.91 nm and 378.10 ± 10.13 nm, respectively. The results indicated that CHI modification increases the average size of the nanoliposomes, which was attributed to the electrostatic interaction between CHI and the phospholipid bilayer, leading to the formation of a polymer coating on the liposome surface (Tai et al., 2020). This coating not only enhanced the structural stability (as shown in EE results) but also improved colloidal dispersion in aqueous media. The PDI of DMY-Lips and CHI-DMY-Lips were 0.24 ± 0.01 and 0.18 ± 0.02, respectively (Table 1), indicating that the liposome particles exhibit a uniform size distribution and suggesting a narrower particle size distribution in CHI-DMY-Lips (see the inset of Fig. 1**B and D**).

As shown in Table 1, DMY-Lips exhibited a negative charge of −19.80 ± 0.90 mV due to ionized phospholipid head groups, whereas CHI-DMY-Lips displayed a positive zeta potential of +23.50 ± 1.02 mV, similar to the observations by Ding et al. (2024), which confirmed the successful coating of CHI on the liposome surface through charge reversal. The positive surface charge of CHI-DMY-Lips may facilitate improved interaction with negatively charged biological membranes, potentially enhancing cellular uptake and bioavailability (Fathima et al., 2022). The CHI layer may also contribute to mucoadhesive properties, beneficial for targeted delivery applications (Shi et al., 2025).

The morphological characteristics of DMY-Lips and CHI-DMY-Lips were investigated. At the macroscopic level, both samples presented as homogeneous, milky-white translucent solutions without phase separation (Fig. 1A and C). The colloidal property of these solutions was verified by the distinct Tyndall effect under laser illumination. TEM imaging (Fig. 1B and D) revealed that both samples consisted of well-dispersed, spherical vesicles with uniform dimensions and intact morphologies, exhibiting no signs of aggregation or rupture (Li et al., 2021). Notably, a thin coating layer of CHI was observed on the surface of the vesicles in CHI-DMY-Lips, while the core vesicular structure remained intact (Fig. 1D). These findings suggest that DMY-Lips and CHI-DMY-Lips possess the typical morphology, homogeneity, and integrity required for a liposomal delivery system. Among them, CHI-DMY-Lips exhibit superior nanocarrier performance due to optimized physicochemical properties.

### 3.2 Characterization and bioactivity of DMY-Lips and CHI-DMY-Lips

#### 3.2.1 UV–Vis spectroscopy analysis

As shown in Fig. 1E, free DMY displayed a characteristic absorption peak at 290 nm, which was attributed to the π → π* transition of the cinnamoyl system in its B ring (Guo et al., 2012). After being encapsulated into liposomes, this peak underwent a blue shift to 325 nm (DMY-Lips). This shift arises from the altered microenvironment within the hydrophobic lipid bilayer, which modifies the electron distribution in DMY's conjugated system, thereby reducing its effective polarity and conjugation (Wei et al., 2025). Further coating with CHI caused a red shift of the peak back to 290 nm (CHI-DMY-Lips). This is probably due to the potential intermolecular interactions (*e.g.*, electrostatic and hydrogen bonding interactions) between the functional groups of CHI and the phenolic hydroxyl groups of DMY. These interactions modulate the electronic properties of DMY's chromophore, leading to the observed spectral recovery (Alomrani et al., 2019). Additionally, no characteristic absorption was observed in CHI-Blank-Lips between 200 and 450 nm, confirming that there was no spectral interference from the carriers.

#### 3.2.2 FTIR spectroscopy analysis

FTIR spectroscopy is widely used to characterize the structure and surface functional groups of nanoparticles. As shown in Fig. 1F, the peaks observed at 3350 cm^−1^ for DMY, 3450 cm^−1^, 3439 cm^−1^, and 3450 cm^−1^ for the liposomal samples (CHI-DMY-Lips, DMY-Lips, and CHI-Blank-Lips), and 3433 cm^−1^ for CHI, respectively, correspond to the O—H stretching vibration of hydrogen bonds, and non‑hydrogen bonded N—H stretching, and free O—H stretching, which suggests the establishment of a hydrogen-bonding network (Hashemi et al., 2017). In the liposomal samples, the peaks at 2926 cm^−1^ and 2857 cm^−1^ are ascribed to the asymmetric and symmetric stretching vibrations of —CH₂ groups, mainly originating from the hydrophobic fatty acid chains within the lipid bilayer (Hu et al., 2025). The absence of peak shifts after CHI coating suggests that the modification occurs on the liposome surface without disrupting the internal hydrophobic structure (Sun et al., 2021). Moreover, the absorptions at 1736 cm^−1^ and 1164 cm^−1^ confirm the existence of C=O and C—O—C groups in the liposomal samples. In the CHI-DMY-Lips spectrum, the characteristic peaks of CHI were preserved, including those at 1636 cm^−1^ (amide I) and 1380 cm^−1^ (athe CHIe III). However, the N—H bending vibration peak of CHI at 1599 cm^−1^ vanished in CHI-DMY-Lips spectrum, suggesting that CHI is bound to the liposome surface through electrostatic interactions (Khoerunnisa et al., 2021). For pure DMY, characteristic peaks were observed at 1641 cm^−1^ (C=O), 1550 cm^−1^ (C=C), 1356 cm^−1^ (C—OH), and 1028 cm^−1^ (C—O—C) (Jiang et al., 2026). In the CHI-Blank-Lips spectrum, the peaks at 1078 cm^−1^ and 864 cm^−1^ correspond to the symmetric stretching vibration of PO₂^−^ and the asymmetric stretching vibration of –N^+^(CH₃)₃ in phospholipids, respectively (Sarabandi & Jafari, 2020). Significantly, all the characteristic peaks of DMY were absent in DMY-Lips and CHI-DMY-Lips, which verifies that DMY was completely encapsulated within the liposomal bilayers without notable chemical interaction with the phospholipids.

#### 3.2.3 *In vitro* antioxidant activity

**Fig. 2:**
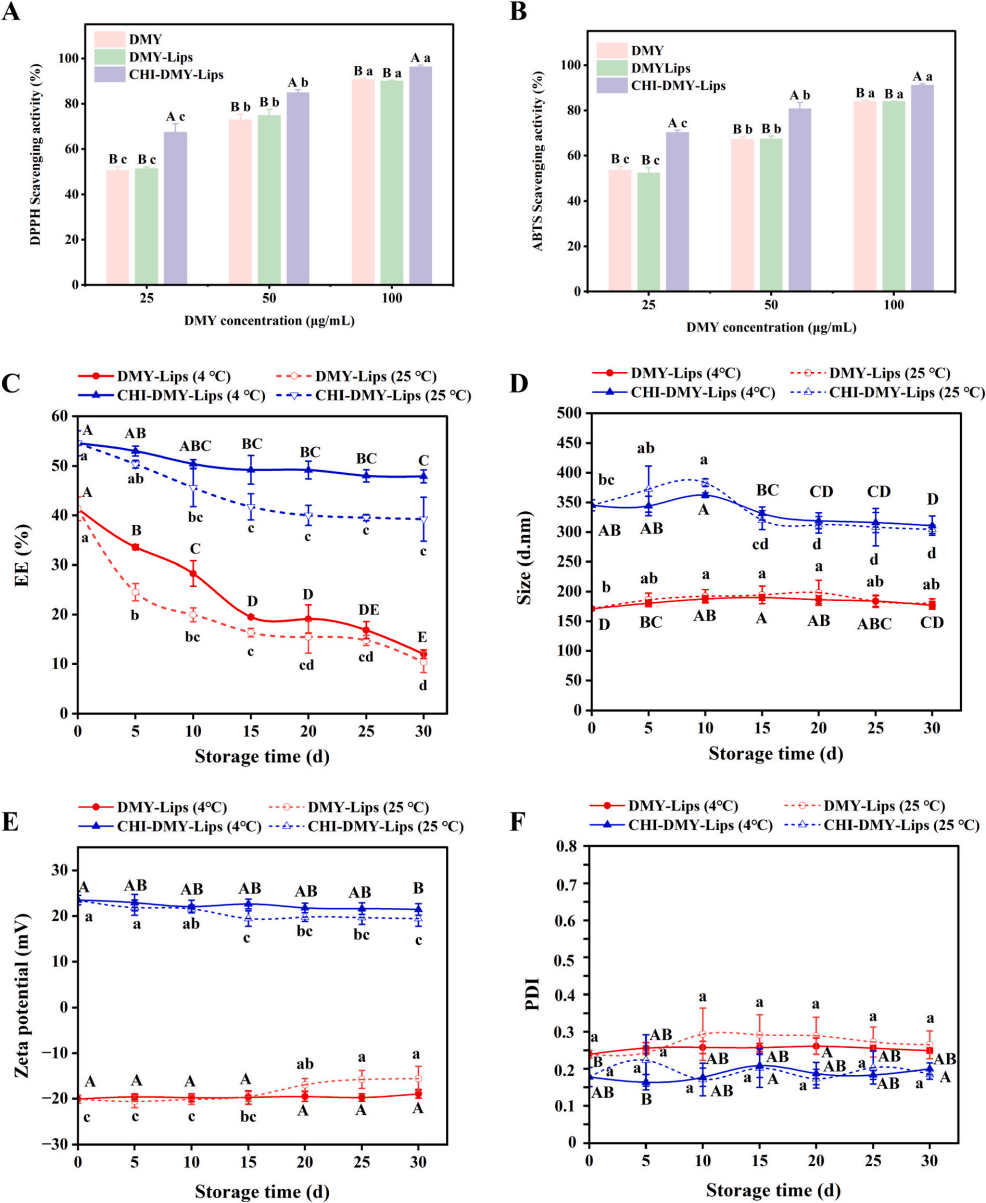
Antioxidant activity and storage stability of dihydromyricetin liposomes (DMY-Lips) and chitosan-modified DMY liposomes (CHI-DMY-Lips). (A) DPPH (2,2-diphenyl-1-picrylhydrazyl) radical scavenging activity; (B) ABTS (2,2′-azino-bis (3-ethylbenzothiazoline-6-sulfonic acid) radical scavenging activity; (C–F) Changes in encapsulation efficiency (EE), mean particle size, zeta potential, and polydispersity index (PDI) of both formulations during storage at 4 °C and 25 °C over different durations. Different letters indicate significant differences among the data for the same sample after different storage durations (*n* = 3, *p* < 0.05).

The antioxidant activities of DMY and its liposomal formulations at varying concentrations (25, 50, and 100 μg/mL) were evaluated using the DPPH and ABTS radical scavenging assays. As shown in Fig. 2A, within the DPPH assay, the scavenging rates of all three formulations exhibited a gradual increase with the rise in concentration. At a concentration of 100 μg/mL, CHI-DMY-Lips achieved the highest scavenging rate of 96.40 ± 0.74%, significantly surpassing those of DMY (90.73 ± 0.55%) and DMY-Lips (90.05 ± 0.33%) at the same concentration (*p* < 0.05). Likewise, in the ABTS assay (Fig. 2B), CHI-DMY-Lips consistently demonstrated superior scavenging ability across all the concentrations under investigation, reaching 91.21 ± 0.66% at 100 μg/mL, which was significantly higher than those of both DMY (84.01 ± 0.49%) and DMY-Lips (83.91 ± 0.13%) (p < 0.05). Remarkably, CHI-coated CHI-DMY-Lips demonstrated significantly enhanced antioxidant effects at all concentrations in comparison to free DMY and uncoated liposomes (p < 0.05), implying a potential synergistic interaction between CHI and DMY (Esposto et al., 2021). These results indicate that the liposomal systems maintain DMY's antioxidant activity, while CHI surface modification further enhances its efficacy, confirming CHI-DMY-Lips as a promising functionalized delivery platform for DMY.

#### 3.2.4 Storage stability analysis

The storage stability of DMY-Lips and CHI-DMY-Lips was assessed over a period of 30 days at 4 °C and 25 °C through the monitoring of EE, particle size, PDI, and zeta potential (Fig. 2**C—F**). Over the 30-day storage period, despite the final EE values being comparable, the decline rate of EE of DMY-Lips at 4 °C was notably lower than that at 25 °C (particularly within the initial fifteen days), which implies that low temperature effectively suppressed drug leakage (Fig. 2C). CHI modification further improved the encapsulation stability, as CHI-DMY-Lips exhibited higher drug retention under identical conditions, indicating that the surface coating could retard the release of encapsulated substances through steric hindrance and molecular interactions (Hu et al., 2025). This stabilizing effect is consistent with the findings of Wang et al. (2025), who reported that CHI-coated liposomes loaded with ACE-inhibitory peptides maintained significantly higher EE than uncoated liposomes after 30 days of storage at 25 °C, and with the observations of Li et al. (2024), who found that CHI coating improved clove essential oil retention in double-loaded liposomes during storage.

Particle size analysis revealed that DMY-Lips exhibited a gradual increase in size during the initial 15-day period at 4 °C, and this increase was more evident during the initial 20-day period at 25 °C (Fig. 2D). This phenomenon can be attributed to the aggregation and fusion of phospholipids, as phospholipid membranes tend to undergo structural reorganization during storage, leading to vesicle coalescence, which becomes more pronounced at higher temperatures (Mukhina et al., 2022). A slight decrease in size was observed between the 20th and 30th days, potentially related to membrane structural changes induced by phospholipid degradation (Tai et al., 2020). Regarding CHI-DMY-Lips, a minor increment in size occurred within the first 0–10 days. This might be attributed to the continuous adsorption of CHI on the liposomal surface, as a higher temperature is more conducive to the adsorption. Nevertheless, it decreased during the period from 15 to 30 days (particularly under 25 °C), indicating a partial dissociation between CHI and the liposome surface (Li et al., 2024). This trend of CHI-coated liposomes first increasing and then decreasing in size during storage is also consistent with the observations of Tai et al. (2020). During the 0–30 days period, the PDI maintained a relatively stable state (no significant differences were observed), suggesting that the particle size of the liposomal samples exhibits a uniform distribution throughout the storage process.

Zeta potential results demonstrated that DMY-Lips maintained a stable potential at 4 °C during the entire storage period. In contrast, a decrease in the absolute value of the potential was detected after 15 days at 25 °C (Fig. 2E). This could potentially be due to the accumulation of phospholipid degradation products, which leads to reduced liposomal stability. CHI-DMY-Lips displayed a stable potential ranging from +21 to +23 mV over a 25-day period at 4 °C, with a significant change occurring by the 30th day (*p* < 0.05). At 25 °C, a significant decline was observed as early as the 15th day (p < 0.05), indicating that the CHI layer is more prone to swelling or ion exchange at higher temperatures (Wang et al., 2025). These findings indicate that both low-temperature storage and CHI coating play a role in maintaining the electrostatic and colloidal stability of the liposomal systems.

### 3.3 *In vitro* release analysis

**Fig. 3:**
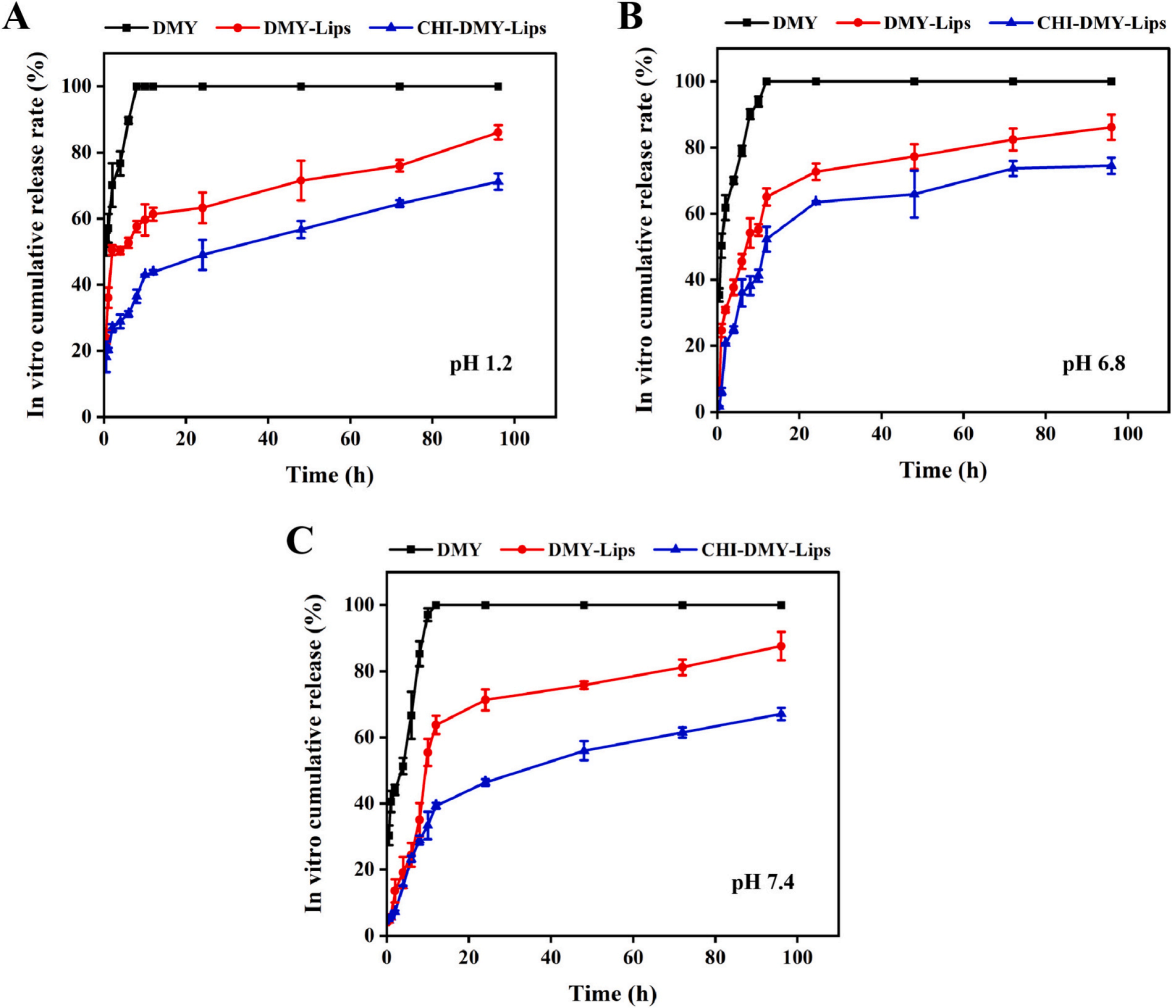
The cumulative release profiles of dihydromyricetin (DMY) from free DMY, dihydromyricetin liposomes (DMY-Lips), and chitosan-modified dihydromyricetin liposomes (CHI-DMY-Lips) in phosphate-buffered saline (PBS) at three pH values: (A) pH 1.2 simulated gastric environment, (B) pH 6.8 intestinal medium, and (C) pH 7.4 intestinal medium.

The *in vitro* cumulative release rate of DMY was calculated and plotted as a function of time for non-encapsulated DMY and the liposomal samples (Fig. 3). As depicted in the graphs, free DMY was rapidly and completely released within 8 h at pH 1.2, and within 12 h at both pH 6.8 and pH 7.4. In comparison, both DMY-Lips and CHI-DMY-Lips demonstrated notable sustained-release effects without burst release. The overall release rate of CHI-DMY-Lips was significantly lower than that of DMY-Lips in the three release media, indicating that the CHI coating might form a diffusion barrier by increasing membrane thickness and hydrophilicity, thus delaying drug release (Imam et al., 2021). Table 2 presents the fitted equations and correlation coefficients of each release profile, which illustrate the release patterns of different DMY delivery forms. The release of free DMY in the three media conforms to the first-order kinetic model (R^2^ values are 0.752, 0.849, and 0.866, respectively), indicating that its release is mainly controlled by the drug's intrinsic dissolution rate and concentration-gradient diffusion, displaying typical rapid-release characteristics (Costa & Lobo, 2001). Liposome encapsulation notably altered the release behavior of DMY and exhibited distinct pH-dependent properties. In an acidic medium with a pH of 1.2, the release of both DMY-Lips and CHI-DMY-Lips was most appropriately described by the Ritger-Peppas model, with release exponent (n) values below 0.43. This suggests that the release is predominantly governed by the Fickian diffusion mechanism, in which the diffusion of drug molecules across the lipid bilayer serves as the rate-limiting step (Ritger & Peppas, 1987). Nevertheless, under conditions of pH 6.8 and 7.4, the release of both liposomes still conformed best to the first-order kinetic model, yet the release constants were significantly lower in comparison to free DMY. CHI modification further exhibits a clear enhancement of the slow-release effect while maintaining the aforementioned pH-dependent release pattern. Under all corresponding pH conditions, the n values of CHI-DMY-Lips are lower than those of DMY-Lips, which reflects the continuous equilibrium process of DMY from the interior of the liposomes to the medium. This confirms that the CHI coating effectively delays drug release by establishing an additional hydrophilic barrier (Zhou et al., 2018). Importantly, both liposomes exhibited the slowest release rates in a physiological environment with a pH of 7.4, suggesting good biocompatibility and potential advantages for intestinal sustained-release. In summary, liposomal encapsulation could achieve the transition from dissolution-controlled release to pH-dependent diffusion release of DMY. Moreover, CHI modification can further strengthen the sustained-release effect, providing a crucial foundation for the design of a stable and predictable release behavior of DMY oral delivery systems.

**Table 2:** The fitted release kinetics equations and correlation coefficients (R^2^) for free DMY, dihydromyricetin liposomes (DMY-Lips) and chitosan-modified dihydromyricetin liposomes (CHI-DMY-Lips).^a^

Sample	Dissolution medium	Digital model	Equation	R^2^
DMY	pH 1.2	First-order kinetics equation	M_t_ = 96.39 (1 − e^−0.90t^)	0.752
	pH 6.8		M_t_ = 95.32 (1 − e^−0.55t^)	0.849
	pH 7.4		M_t_ = 99.89 (1 − e^−0.25t^)	0.866
DMY-Lips	pH 1.2	Ritger-Peppas	M_t_ = 43.86 x^0.18^	0.899
	pH 6.8	First-order kinetics equation	M_t_ = 79.20 (1 − e^−0.15t^)	0.932
	pH 7.4		M_t_ = 82.86 (1 − e^−0.09t^)	0.961
CHI-DMY-Lips	pH 1.2	Ritger-Peppas	M_t_ = 24.82 x^0.29^	0.983
	pH 6.8	First-order kinetics equation	M_t_ = 71.01 (1 − e^−0.10t^)	0.978
	pH 7.4		M_t_ = 61.81 (1 − e^−0.07t^)	0.981

a First-order kinetics equation: M_t_ = M_∞_ (1 − e^−k·t^); Ritger-Peppas: M_t_ = k·t^n^. M_t_ represents the cumulative release percentage at time t (h), M_∞_ represents the cumulative release amount at infinite time, and k represents release constant.

### 3.4 *In vitro* simulated digestive stability and bioaccessibility

#### 3.4.1 Changes in particle size, PDI, and zeta potential during *in vitro* digestion

**Fig. 4:**
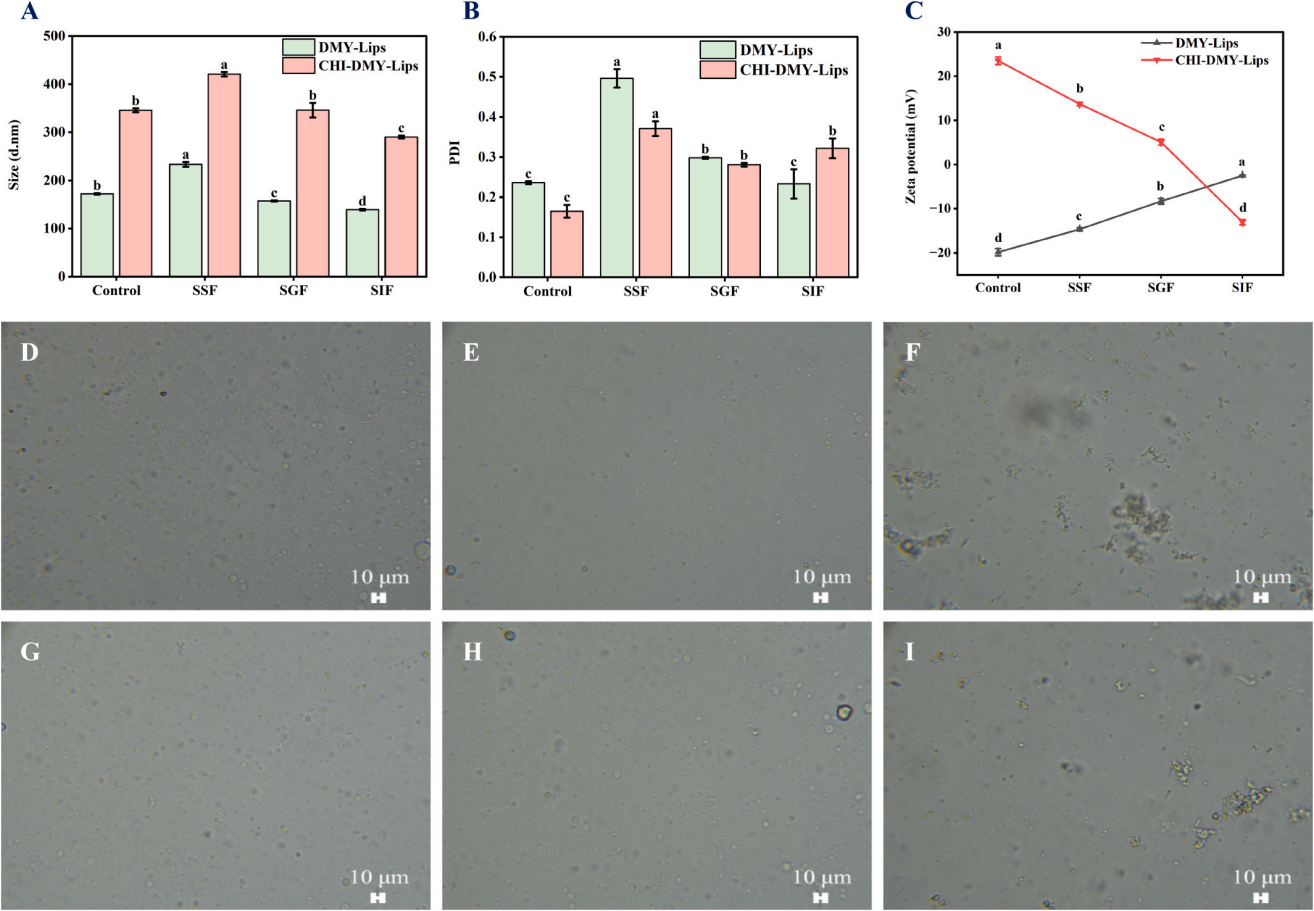
Dynamic light scattering (DLS) characterization and morphological observation of two liposomal formulations during *in vitro* simulated digestion. SSF = simulated salivary fluid (oral phase), SGF = simulated gastric fluid (gastric phase), SIF = simulated intestinal fluid (intestinal phase). (A–C) Particle size, polydispersity index (PDI) and zeta potential of dihydromyricetin liposomes (DMY-Lips) and chitosan-modified dihydromyricetin liposomes (CHI-DMY-Lips) after SSF, SGF, SIF incubation; (D–F) Bright-field images of DMY-Lips after oral, gastric, and intestinal digestion; (G–I) Bright-field images of CHI-DMY-Lips after oral, gastric, and intestinal digestion.

Under simulated GI digestion conditions, the stability profiles of liposomes were evaluated by monitoring changes in their physicochemical properties, as illustrated in Fig. 4**A–C**. In the oral digestion phase, both DMY-Lips and CHI-DMY-Lips exhibited significant increases in particle size and PDI (Fig. 4A and B). This indicates a reduction in the homogeneity of the colloidal dispersion, which is consistent with previous reports on liposomal size enlargement after oral digestion (Ren et al., 2024). The increase of particle size in DMY-Lips could be ascribed to the electrostatic neutralization between cations (*e.g.*, K^+^, Mg^2+^) present in the oral digestive fluid and the negatively charged groups of phospholipids. In the case of CHI-DMY-Lips, it likely results from the structural instability of CHI under neutral conditions, leading to particle aggregation. Concurrently, the adsorption of ions such as Na^+^ and CO₃^2−^ neutralizes the surface charge of the liposomal particles, resulting in a reduction of the absolute zeta potential values in both systems (Fig. 4C). After gastric digestion, notable decreases in the mean particle size were observed for both DMY-Lips and CHI-DMY-Lips (Fig. 4A). This phenomenon is primarily related to the weakened electrostatic repulsion under low pH conditions and the osmotic pressure differences across the augmented lipid bilayer (Liu et al., 2020). Notably, DMY-Lips showed a more pronounced reduction in size compared to CHI-DMY-Lips, which maintained their pre-digestion particle size level after gastric exposure, indicating that CHI modification effectively mitigated the impacts of the gastric environment on the structural stability and integrity of liposomes. Similarly, within the acidic and high ionic strength environment of gastric digestion, H^+^ and NaHCO_3_^−^ neutralize the surface charge of liposomes, leading to a decrease in the absolute zeta potential values of DMY-Lips and CHI-DMY-Lips after 2 h of gastric digestion (Fig. 4C). In the intestinal phase, both liposomes exhibited a further decrease in particle size (Fig. 4A), which was mainly attributed to the hydrolysis of the phospholipid bilayer by pancreatic enzymes and the structural alterations mediated by bile salts. This reduction in particle size may have a positive correlation with the absorption efficiency of small intestinal epithelial cells, potentially promoting DMY absorption (Ji et al., 2017). Furthermore, after intestinal digestion, both formulations displayed negative zeta potentials (Fig. 4C). This phenomenon may stem from the hydrolysis of phospholipids by pancreatic enzymes, resulting in the formation of polar anionic products (*e.g.*, lysophospholipids and free fatty acids), which interact with the liposomal surface, thereby causing a redistribution of surface charge (Pan et al., 2022).

#### 3.4.2 Changes in liposomal morphology during *in vitro* digestion

The structural changes of DMY-Lips and CHI-DMY-Lips in the simulated GI environment were evaluated by bright-field microscopy (Fig. 4**D–I**). During the oral and gastric digestion stages (Fig. 4D, E, G, and H), both liposomes exhibit intact spherical vesicle structures. This observation aligns with the general consensus, as phospholipids cannot be hydrolyzed by gastric lipase, and the well-organized lipid bilayer provides resistance against gastric environmental stress (Liu et al., 2020). Furthermore, the decrease in liposome particle size observed during these stages (as previously described) is not due to structural disintegration but rather to physical compaction triggered by low pH (*e.g.*, alterations in osmotic pressure and reduction in electrostatic repulsion resulting in densification), a phenomenon consistent with previous reports on the behavior of liposomes in simulated gastric fluid (Liu et al., 2020). However, during the intestinal digestion stage (Fig. 4F and I), significant morphological differences were observed between the two liposomes. DMY-Lips exhibited near-total disintegration, with intact vesicles hardly identifiable in the field of view, indicating that their structure was likely thoroughly degraded by bile salts and pancreatic enzymes. In contrast, a considerable number of structurally intact liposomes were still observable for CHI-DMY-Lips. These results visually demonstrate that the CHI modification layer effectively delayed the erosion of liposomes by intestinal digestive fluids, allowing them to maintain structural integrity for a longer period in the small intestine (the primary absorption site). This provides direct structural stability evidence for the enhanced DMY delivery efficiency of CHI-DMY-Lips in intestinal environments.

#### 3.4.3 Changes in DMY concentration during *in vitro* digestion

**Fig. 5:**
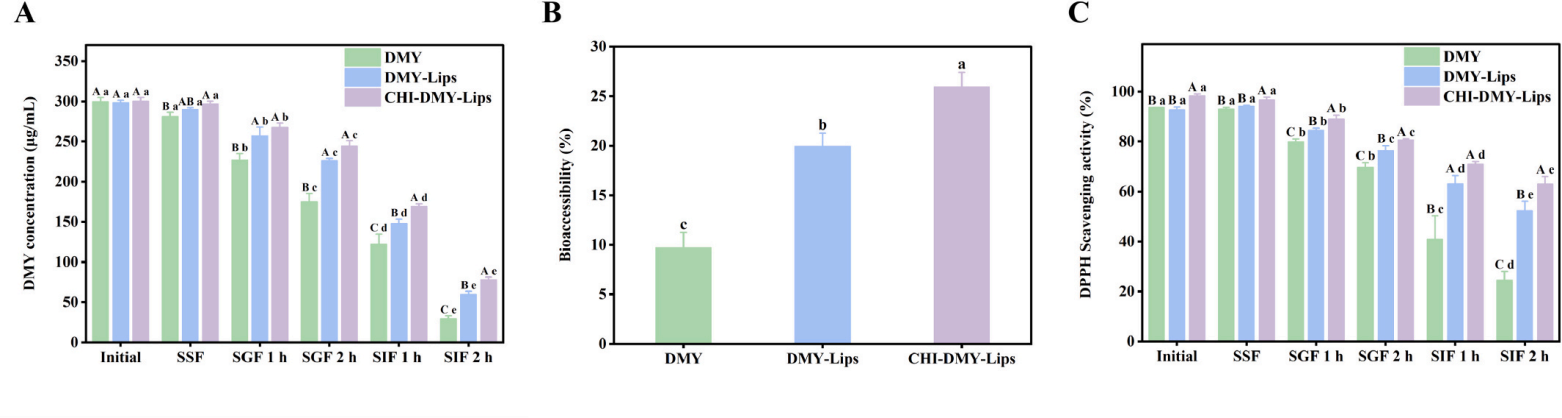
Changes in (A) residual dihydromyricetin (DMY) concentration, (B) bioaccessibility, and (C) DPPH (2,2-diphenyl-1-picrylhydrazyl) radical scavenging activity of dihydromyricetin liposomes (DMY-Lips) and chitosan-modified dihydromyricetin liposomes (CHI-DMY-Lips) after sequential simulated salivary fluid (SSF), simulated gastric fluid (SGF), and simulated intestinal fluid (SIF) digestion stages.

As shown in Fig. 5A, at the SSF stage, no statistically significant differences were observed in DMY concentration of the three systems compared to pre-digestion levels. The brief oral digestion duration and the lack of specific enzymes resulted in a weak direct impact on the liposomal structure, consequently failing to trigger significant release of the encapsulated DMY (Song et al., 2023). However, the DMY concentration of the three systems decreased significantly after 1 h of gastric digestion, and the free DMY group was significantly lower than both of the two liposome systems (*p* < 0.05). This can mainly be attributed to the direct exposure of free DMY to the gastric environment, in which its flavonoid structure is susceptible to acid-catalyzed hydrolysis and oxidative degradation (Mei et al., 2025). In contrast, the phospholipid bilayer of liposomes serves as a physical barrier, effectively minimizing the direct contact between DMY and the acidic gastric environment. After 2 h of gastric digestion, the DMY concentration in CHI-DMY-Lips was obviously higher than that in DMY-Lips, indicating that CHI modification offers better protective effects in the later stages of gastric digestion. This may be attributed to the protonation of amino groups in CHI under acidic conditions, which enhances its interaction with the liposomal surface and further mitigates the adverse effects of gastric acid on the structural integrity of liposomes (consistent with the results of particle size changes) (Li et al., 2024). At the end of intestinal digestion, significant differences in DMY concentration were observed among the three systems (p < 0.05). Free DMY degraded rapidly because it was directly exposed to the intestinal environment, whereas liposomal encapsulation significantly slowed down this process. Nevertheless, the hydrolysis of the phospholipid bilayer by bile salts and pancreatic enzymes still resulted in the gradual release of DMY (Song et al., 2023). The CHI layer on the surface of CHI-DMY-Lips further mitigated the erosion of the carrier structure by digestive enzymes through steric hindrance and adhesion effects, thereby achieving better controlled release and protection of DMY.

#### 3.4.4 The bioaccessibility of DMY

Bioaccessibility refers to the proportion of bioactive components capable of being absorbed by the human body. This concept holds significant importance in the evaluation of the nutritional value of foods and their formulations, particularly in offering valuable guidance for the development of food systems designed to promote human health. As shown in Fig. 5B, the bioaccessibility of DMY was significantly enhanced by liposomal encapsulation, increasing from 9.73 ± 1.51% for free DMY to 19.98 ± 1.31% for DMY-Lips, and further to 25.97 ± 2.44% for CHI-DMY-Lips. This value is comparable to that reported for other advanced DMY delivery systems, such as egg yolk phospholipid peptide-DMY nanocomposites (24.97%) (Wei et al., 2025) and DMY/soy protein isolate Pickering emulsions (33.51%) (Mei et al., 2025), both of which employed a similar micelle-based bioaccessibility determination method. Notably, our CHI-DMY-Lips achieved a 2.67-fold higher bioaccessibility than free DMY, demonstrating the effectiveness of the CHI-coated liposomal system in enhancing the potential absorbable fraction of this flavonoid. In light of the previous analysis on the stability of liposomes and the changes in DMY concentration during the digestion process, the liposomal structure can reduce the premature release of DMY and effectively protect it from degradation in the GI environment, while CHI modification further delays the release of DMY and promotes its absorption by enhancing carrier stability and intestinal adhesion.

#### 3.4.5 Changes of antioxidant capacity during *in vitro* digestion

The antioxidant activity of DMY is closely related to its chemical structure. Different GI environments may alter its structural stability, thereby directly affecting its final antioxidant capacity (Xiang et al., 2017). As shown in Fig. 5C, during the oral digestion stage, all groups maintained high DPPH scavenging activity, likely due to the short digestion time that did not yet induce significant degradation or structural changes in DMY. As digestion progressed into the gastric and intestinal stages, the antioxidant activity of all samples gradually decreased, but liposomal encapsulation significantly delayed this decline. In the gastric digestion stage, the scavenging rate of CHI-DMY-Lips (80.54%) was significantly higher than that of DMY-Lips (76.31%) and free DMY (69.63%) (*p* < 0.05). By the end of intestinal digestion, CHI-DMY-Lips still maintained the highest scavenging rate (62.94%, significantly surpassing the other two groups, p < 0.05), suggesting that CHI-DMY-Lips may mitigate oxidative stress in metabolic disorders. This trend is consistent with the change in DMY concentration during digestion, indicating that liposomal encapsulation and CHI modification protect the antioxidant activity of DMY by delaying its release and maintaining its effective concentration during GI digestion.

## 4 Conclusion

This research successfully developed CHI-DMY-Lips as an advanced oral delivery system with enhanced stability, activity, and bioaccessibility. Electrostatic interactions, hydrogen bonding, and hydrophobic interactions jointly promote the formation and stabilization of CHI-DMY-Lips. In this process, CHI is predominantly driven by electrostatic interactions to adsorb onto the negatively charged liposomal surface, resulting in the generation of positively charged colloidal particles with larger size, more uniform distribution, and higher EE (54.00% compared to 41.01% in DMY-Lips). The encapsulation by liposomes preserves the antioxidant activity of DMY, while CHI further augments its activity through synergistic effects. During a 30-day storage period, CHI-DMY-Lips showed enhanced structural and electrostatic stability (with smaller changes in EE and zeta potential values). Moreover, CHI-DMY-Lips demonstrated superior *in vitro* sustained-release performance (transitioning from dissolution-controlled release to pH-dependent diffusion release), with the optimal sustained-release effects being observed at pH 7.4. Most crucially, CHI-DMY-Lips retained structural integrity throughout GI digestion and protected DMY from acid/enzyme-induced degradation. It achieved a bioaccessibility approximately threefold that of free DMY (25.97% *vs.* 9.73%), while preserving its effective concentration and activity. These findings validate the significance of CHI-DMY-Lips as an oral delivery system that combines efficient drug delivery with functional preservation. Despite these encouraging findings, the *in vivo* oral bioavailability and pharmacokinetics of CHI-DMY-Lips remain to be elucidated in animal models. Further studies on long-term storage stability and industrial scalability are also needed to advance this system toward practical applications in functional foods and nutraceuticals.

## CRediT authorship contribution statement

**Hui Teng:** Supervision, Project administration, Investigation, Conceptualization. **Minyi Yan:** Writing – original draft, Methodology, Investigation, Formal analysis, Data curation. **Chong Chen:** Writing – review & editing, Visualization, Supervision, Funding acquisition, Conceptualization. **Chao Ai:** Writing – review & editing, Validation, Software, Project administration. **Huangjuan Huo:** Validation, Software, Methodology, Investigation. **Lei Chen:** Visualization, Resources, Formal analysis, Data curation.

## Declaration of competing interest

The authors declare that they have no known competing financial interests or personal relationships that could have appeared to influence the work reported in this paper.

## Data Availability

Data will be made available on request.
